# Expression of the Testis-Specific Serine/Threonine Kinases Suggests Their Role in Spermiogenesis of Bay Scallop *Argopecten irradians*

**DOI:** 10.3389/fphys.2021.657559

**Published:** 2021-03-30

**Authors:** Xinru Xue, Lingling Zhang, Yajuan Li, Huilan Wei, Shaoxuan Wu, Tian Liu, Liangjie Liu, Qiang Xing, Shi Wang, Zhenmin Bao

**Affiliations:** ^1^MOE Key Laboratory of Marine Genetics and Breeding, Ocean University of China, Qingdao, China; ^2^Laboratory for Marine Fisheries Science and Food Production Processes, Pilot National Laboratory for Marine Science and Technology (Qingdao), Qingdao, China; ^3^Laboratory for Marine Biology and Biotechnology, Pilot National Laboratory for Marine Science and Technology (Qingdao), Qingdao, China; ^4^Laboratory of Tropical Marine Germplasm Resources and Breeding Engineering, Sanya Oceanographic Institution, Ocean University of China, Sanya, China

**Keywords:** serine/threonine kinases, testis-specific expression, Tssk family, spermiogenesis, scallop

## Abstract

Members of the testis-specific serine/threonine kinases (Tssk) family play critical roles in spermatogenesis in vertebrates. But in mollusks, research on Tssk family is still lagging. In this study, we systematically identified Tssk family based on the genomic and transcriptomic data from a commercially important scallop *Argopecten irradians* and detected the spatiotemporal expression in adult gonads. Five members were identified, with the gene length varying from 1,068 to 10,729 bp and the protein length ranging from 294 to 731 aa. All the Tssks possess a serine/threonine protein kinase catalytic (S_TKc) domain. Phylogenetic analysis revealed existence of four homologs of vertebrate Tssk1/2, Tssk3, Tssk4, Tssk5, and absence of Tssk6 in the scallop. The remaining gene (Tssk7) formed an independent clade with Tssks of other mollusks and arthropods, indicating that it may be a new member of Tssk family unique to protostomes. By investigating the expression of *Tssks* in four developmental stages of testes and ovaries, we found all five *Tssks* were primarily expressed in mature testis. *In situ* hybridization experiment revealed the five *Tssks* were localized in the spermatids and spermatozoa. The testis-predominant expression of Tssk family suggests Tssks may play pivotal roles in spermiogenesis in the scallop. Our study provides basic information on the characteristics and expression profiles of Tssk family of *A. irradians*. To our knowledge, it represents the first comprehensive analysis of Tssk family in mollusks.

## Introduction

Spermatogenesis is a complicated but well-organized process that universally exists in the animal kingdom. It can be divided into three main phases: (1) proliferative phase, in which spermatogonia undergo mitotic division and produce a large number of spermatocytes; (2) meiotic phase, in which haploid spermatids are generated; and (3) spermiogenesis phase, in which spermatids differentiate into spermatozoa. The whole process requires strict gene expression and regulation, and spermatogenic failure results in male infertility with azoospermia or oligozoospermia (Cannarella et al., 2019). As one of the most important and universal ways of post-translational modification, protein phosphorylation plays a prominent role in spermatogenesis. A large number of serine/threonine kinases and tyrosine kinases have been demonstrated to be expressed at various stages of sperm development (Jenardhanan and Mathur, 2014). Study on these kinases can help to understand the machinery of spermatogenesis.

In recent years, a family of testis-specific protein kinases, called as testis-specific serine/threonine kinases (Tssks), has attracted researcher’s interest due to their essential roles in spermatogenesis (Wang et al., 2015a; Li et al., 2016; Kim et al., 2019). Six Tssk subfamilies have been reported in mammals, including Tssk1 through Tssk6, which have the conserved serine/threonine protein kinases catalytic (S_TKc) domain (Wang et al., 2015a). Most of them are specifically expressed in spermatids or sperm (Li et al., 2011) and have been demonstrated to play essential roles in spermatogenesis. For example, *Tssk1* and *Tssk2* double-deletion resulted in male sterility in mice (Xu et al., 2008; Shang et al., 2010). *Tssk6* gene deletion resulted in a male infertile phenotype caused by certain morphological defects in the sperm (Spiridonov et al., 2005; Sosnik et al., 2009), and knockout of *Tssk4* produced a subfertility phenotype in mice due to severely reduced sperm motility (Wang et al., 2015b). Therefore, Tssk family plays a fundamental role in spermiogenesis in mammals.

Many mollusks are important aquaculture species such as oysters, scallops, and mussels. For a precise reproductive control during the breeding process, a comprehensive understanding of molecular mechanism underlying gametogenesis is required. Massive screening of reproduction-specific genes in scallop *Argopecten purpuratus* (Boutet et al., 2008), mussel *Mytilus edulis* (Ciocan et al., 2011), and clam *Tridacna squamosa* (Li et al., 2020) revealed that *Tssk* genes are involved in sexual maturation of testis. However, only *Tssk1* has been cloned and characterized in mollusks. In pen shell *Atrina pectinate* (Li et al., 2016), detailed investigation on the expression of *Tssk1* in adult tissues and during male gametogenic cycle revealed the highest level of expression in mature testis, supporting its involvement in the spermatogenesis and/or sperm production in mollusks. A recent study in abalone *Haliotis discus hannai* found a significant reduction of *Tssk1* expression in the triploids than diploids, suggesting that Tssk1 is primarily expressed at the post-meiotic stage and is potentially involved in the sterility and/or partial fertility of male triploidy (Kim et al., 2019). Except for Tssk1, the other Tssk members remain unexplored in mollusks.

The bay scallop (*Argopecten irradians*) is naturally distributed along the Atlantic coast of the United States. Since its first introduction to China in 1982, it has been widely cultivated and becomes one of the most commercially important bivalves in China. Like many marine bivalves, the bay scallop is a hermaphrodite that releases male and female gametes simultaneously, making it difficult to obtain pure oocytes for genetic breeding. Aiming at better understanding and future control of spermatogenesis in simultaneous hermaphrodites, we systematically identified and characterized five *Tssk* genes from the genome and transcriptomes of *A. irradians* and analyzed their temporal and spatial expression patterns in adult gonads. Our study provides basic information on the evolution and function of Tssk family and may contribute to the breeding of some bivalve mollusks.

## Materials and Methods

### Sample Collection and Histological Analysis

To obtain gonads at various gametogenic stages, the bay scallops were collected from Yantai (Shandong Province, China) every month for a year. After being transported to the laboratory, the scallops were acclimated in filtered and aerated seawater for 3 days. About 50 individuals were randomly chosen, and their ovaries and testes were dissected. Parts of them were flash-frozen in liquid nitrogen and stored at −80°C until used for RNA extraction. The rest parts were prepared for paraffin sectioning. They were fixed in 4% paraformaldehyde for 12–24 h followed by washing twice with 1 × PBS, dehydrated in a graded methanol series, and stored at −20°C. Then, the samples were transferred to ethanol, cleared with xylene, embedded in paraffin wax, and cut into 5 μm (ovary) or 3 μm (testis) sections on a rotary microtome (Leica, Wetzlar, Germany). Serial sections were tiled on glass slides, deparaffined with xylene, hydrated with gradient ethanol to water, and stained with hematoxylin. After that, the glass slides were counterstained with eosin, dehydrated with ethanol, cleared with xylene, mounted with neutral balsam, and covered with coverslips. Finally, the sections were observed under a Nikon Eclipse E600 research microscope.

### Identification and Sequence Analysis of the Scallop *Tssk* Genes

The available Tssk protein sequences of representative organisms were downloaded from the NCBI^1^ and UniProt^2^ and then aligned with the bay scallop genome and transcriptomes (Liu et al., 2020) with the E value threshold of 1e-5. The resultant scallop sequences were further confirmed by BLASTX against the non-redundant protein sequences (nr) database. The open reading frame (ORF) of the candidate Tssk sequences was determined using the ORF Finder program.^3^ The conserved domains of the protein sequences were predicted by SMART,^4^ and the structure of the *Tssk* genes was presented by the online software IBS.^5^ The theoretical isoelectric points (PI) and molecular weights (MW) of the Tssk proteins were computed by compute pI/Mw tool.^6^

### Phylogenetic Analysis

To determine which group the *Tssk* genes belong to, phylogenetic analysis was performed. The orthologous Tssk protein sequences of various species were downloaded from NCBI and UniProt. Eighteen organisms were included: mouse (*Mus musculus*), emu (*Dromaius novaehollandiae*), three-toed box turtle (*Terrapene carolina triunguis*), whale shark (*Rhincodon typus*), thorny skate (*Amblyraja radiata*), zebrafish (*Danio rerio*), purple sea urchin (*Strongylocentrotus purpuratus*), crown-of-thorns starfish (*Acanthaster planci*), cotton bollworm (*Helicoverpa armigera*), oriental fruit fly (*Bactrocera dorsalis*), bark scorpion (*Centruroides sculpturatus*), tailed mussel (*Lingula anatina*), Pacific oyster (*Crassostrea gigas*), Yesso scallop (*Mizuhopecten yessoensis*), king scallop (*Pecten maximus*), channeled apple snail (*Pomacea canaliculata*), marsh snail (*Biomphalaria glabrata*), and sea anemone (*Exaiptasia diaphana*). The S_TKc domain was predicted using the online software CDD,^7^ and the serine/threonine protein kinases active site and ATP-binding region were predicted by PROSITE.^8^ Multiple alignments of the S_TKc domains were performed by ClustalW (Larkin et al., 2007), and neighbor-joining (NJ) phylogenetic tree was constructed using MEGA X (Kumar et al., 2018), with a bootstrap value of 1,000.

### RNA Isolation and cDNA Synthesis

Total RNA was isolated using the traditional guanidine isothiocyanate method and digested with DNase I (TaKaRa, Shiga, Japan) to remove residual DNA contamination. RNA concentration and purity were measured with a Nanovue Plus spectrophotometer (GE Healthcare, NJ, United States), and the integrity of RNA was verified by agarose gel electrophoresis. Oligo(dT)_18_ and MMLV reverse transcriptase (TaKaRa, Shiga, Japan) were used to synthesize first-strand cDNA from 2 μg of total RNA in a volume of 20 μl. The reaction was carried out at 42°C for 90 min and terminated by heating at 70°C for 10 min. Finally, the cDNA products were diluted to 10 ng/μl and stored at −20°C until used.

### Quantitative Real-Time PCR

To examine the expression patterns of Tssk family genes in the gonads, quantitative real-time PCR (qRT-PCR) was performed. The gene-specific primers were designed by Primer Premier 5.0, and the primer specificity was tested by alignment with the *A. irradians* genome and transcriptomes using BLASTN with an E value threshold of 1e-10. The primer sequences used for qRT-PCR are listed in Table 1, and elongation factor 1-alpha (EF1A) was used as an endogenous control for normalization of gene expression (Li et al., 2019). Amplification efficiency of each primer pair was calculated based on the standard curve generated from a 2-fold dilution series spanning five orders of magnitude. The qRT-PCR was conducted with the Light Cycler 480 SYBR Green I Master on a Light Cycler 480 Real-time PCR System (Roche Diagnostics, Mannheim, Germany). The PCR program was as follows: 94°C for 10 min, followed by 40 cycles of 94°C for 15 s and 60°C for 1 min. Four samples were assayed for each stage, and all reactions were conducted in triplicate. For each gene, the melting curve was analyzed to confirm that a single PCR product was amplified. The relative expression levels of *Tssk* genes were calculated using the 2^−*Δ*ΔCt^ method. The statistical analysis was performed with SPSS (version 22.0) software using the paired-sample *t*-tests. Values of *p* lower than 0.05 were considered as statistically significant.

**Table 1:** Sequences of primers used for quantitative real-time PCR analysis.

Gene name	Primer sequences (5'-3')	Amplicon length (bp)	Amplification efficiency
Tssk1/2	F:CATTCTATATATTATGGTGTGCGCC R:TGGAGAACCCTATCTTCTTCTCAAG	98	1.03
Tssk3	F:GTGTCCGAGGAATGCCAGAG R:TCTGCGAGCCAGCTGTGAT	101	1.01
Tssk4	F:CAAGTCATTGAGACAACTACACGGT R:GCGACATCCTCCTCTATCATCTT	104	0.97
Tssk5	F:GGAACGAACAAATACCCGGTAG R:TAGATATTCCAGGGCTAGCTGTTG	115	1.02
Tssk7	F:ATGATGTGTGCTACAATGCCCT R:GGCATGTAACTTATCTAATACGCG	111	1.03
EF1A	F:CCATCTGCTCTGACAACTGA R:GGACAATAACCTGAGCCATAA	196	1.02

### *In situ* Hybridization

To determine the localization of *Tssk* genes in mature testis, *in situ* hybridization was performed. First, PCR was conducted using the gene-specific primers (Table 2), and the products were cloned into pMD19-T vector (TaKaRa, Shiga, Japan). Then, the resultant recombinant plasmids were used as templates in the following *in vitro* transcription. Sense and anti-sense digoxigenin-labeled RNA probes were synthesized using DIG RNA labeling mixture (Roche, Mannheim, Germany) and T7/SP6 RNA polymerase (Thermo, Waltham, United States). Meanwhile, the sections of gonadal tissues were serially rehydrated in PBST (PBS plus 0.1% Tween-20) and digested with 2 μg/ml proteinase K at 37°C for 15 min. After prehybridization at 60°C for 4 h, the sections were incubated in hybridization buffer (50% formamide, 5 × SSC, 100 μg/ml yeast tRNA, 1.5% blocking reagent, and 0.1% Tween-20) containing 1 μg/ml denatured RNA probe overnight at 60°C. Then the sections were washed six times at 60°C for 15 min, twice at room temperature for 10 min in maleic acid buffer (0.1 M maleic acid, 0.15 M NaCl, 0.1% Tween-20, and pH 7.5) followed by incubation with alkaline phosphatase-conjugated anti-digoxigenin antibody (Roche, Mannheim, Germany). After extensive washing with PBST, the sections were incubated with NBT/BCIP (Roche, Mannheim, Germany) and counterstained with 1% neutral red. The signals were visualized under a Nikon Eclipse E600 research microscope.

**Table 2:** Sequences of primers used for *in situ* hybridization.

Gene name	Primer sequences (5'-3')
Tssk1/2	F:TCCGTGAAAAGTTCCTTCCTCG R:ACGGGTGCTCTTTGATACTAACGAT F-SP6:ATTTAGGTGACACTATAGTCCGTGAAAAGTTCCTTCCTCG R-T7:TAATACGACTCACTATAGGGACGGGTGCTCTTTGATACTAACGAT
Tssk3	F:TCCTAAGATACATACAGAGAAGCGGG R:TAGCGGTGGAGCGGAACAGT F-SP6:ATTTAGGTGACACTATAGTCCTAAGATACATACAGAGAAGCGGG R-T7:TAATACGACTCACTATAGGGTAGCGGTGGAGCGGAACAGT
Tssk4	F:AGAAGATGATAGAGGAGGATGTCGC R:GCTGGCATTACAGAATCTATGGTTG F-SP6:ATTTAGGTGACACTATAGAGAAGATGATAGAGGAGGATGTCGC R-T7:TAATACGACTCACTATAGGGGCTGGCATTACAGAATCTATGGTTG
Tssk5	F:AGGGGTTATGCGGAGAAAAGG R:TGTCTTCAGGTAGGTGTCACGATTC F-SP6:ATTTAGGTGACACTATAGAGGGGTTATGCGGAGAAAAGG R-T7:TAATACGACTCACTATAGGGTGTCTTCAGGTAGGTGTCACGATTC
Tssk7	F:ATTACCCAACAGCCCGTTTAGATT R:TTGATGGCGTTTCGGTGACTT F-SP6:ATTTAGGTGACACTATAGATTACCCAACAGCCCGTTTAGATT R-T7:TAATACGACTCACTATAGGGTTGATGGCGTTTCGGTGACTT

Primers F and R were used to amplify the cDNA fragment, and the product was used for the second-round PCR with the primers F-SP6 and R-T7.

## Results

### Identification and Sequence Analysis of *Argopecten irradians Tssk* Genes

**Figure 1:**
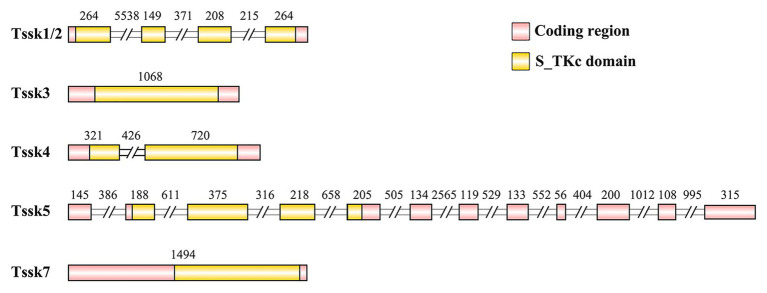
The structure of the *Tssk* genes of *A. irradians*. The pink boxes represent the coding regions, and the yellow boxes indicate the S_TKc domains. The diagram is shown according to the sequence length (bp) except for the intron regions that marked with double slash. The numbers indicate the nucleotide length (bp) of the exons or introns.

In the present study, five members of the Tssk family were identified in the genome of *A. irradians*, which were named as Tssk1/2, Tssk3, Tssk4, Tssk5, and Tssk7. The structure and characteristics of these genes are provided in Table 3 and Figure 1. According to the results, the length of scallop *Tssk* genes varied from 1,068 to 10,729 bp. Two genes (*Tssk3* and *Tssk7*) were intron-free, and the remaining three had 1–11 introns. The length of the five Tssk proteins ranged from 294 to 731 aa, with molecular weight varying from 33.44 to 82.42 kDa.

**Table 3:** Structural characteristics of the five Tssks of *A. irradians*.

Gene name	Gene length (bp)	Exon No.	Protein length (aa)	S_TKc domain length/position (aa)	Molecular weight (kDa)	PI	Accession number
Tssk1/2	7,009	4	294	255/16–270	33.44	9.06	MW273893
Tssk3	1,068	1	355	258/59–316	41.11	9.07	MW273895
Tssk4	1,467	2	346	257/45–301	39.27	9.05	MW273896
Tssk5	10,729	12	731	276/64–339	82.42	9.79	MW273897
Tssk7	1,494	1	497	262/232–493	54.47	8.60	MW273894

**Figure 2:**
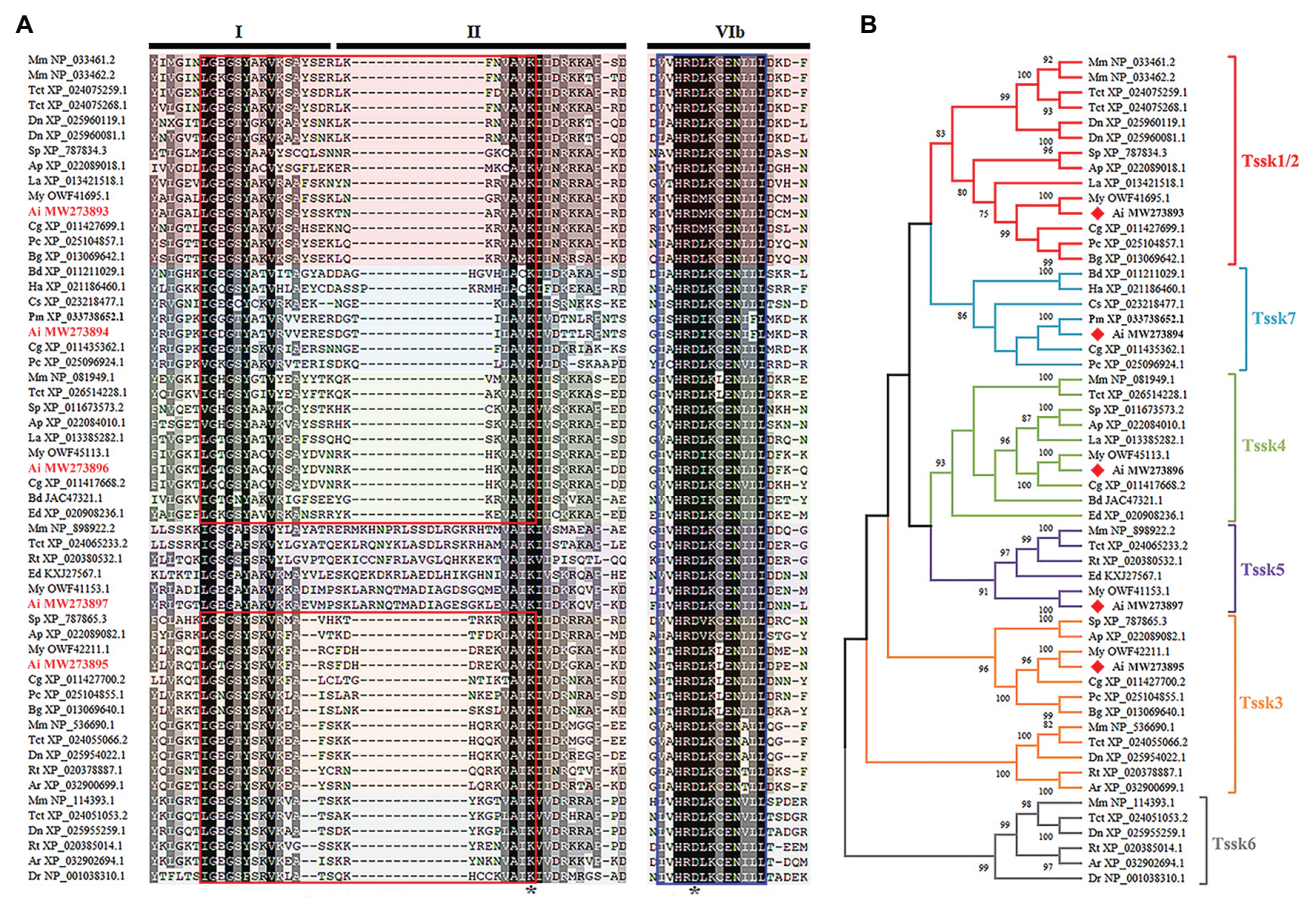
Multiple alignment and phylogenetic analysis of *Tssk* genes. **(A)** The multiple sequence alignment of region I, II, and VIb of Tssk proteins from various species. Identical residues were represented in black and similar residues in gray. The ATP-binding region was marked with a red box and the potential serine/threonine protein kinases active site was marked with a blue box. The asterisks denoted the indispensable residues of lysine and aspartic acids in the S_TKc domain. **(B)** Phylogenetic analysis of the Tssk family genes based on the S_TKc domain. The phylogenetic tree was built using the NJ method in MEGA X software. Tssk proteins of the bay scallop were labeled with red diamonds. The species abbreviations are as follows: *Mus musculus* (Mm), *Dromaius novaehollandiae* (Dn), *Terrapene carolina triunguis* (Tct), *Rhincodon typus* (Rt), *Amblyraja radiata* (Ar), *Danio rerio* (Dr), *Strongylocentrotus purpuratus* (Sp), *Acanthaster planci* (Ap), *Helicoverpa armigera* (Ha), *Bactrocera dorsalis* (Bd), *Centruroides sculpturatus* (Cs), *Lingula anatina* (La), *Crassostrea gigas* (Cg), *Argopecten irradians* (Ai), *Mizuhopecten yessoensis* (My), *Pecten maximus* (Pm), *Biomphalaria glabrata* (Bg), *Pomacea canaliculata* (Pc), and *Exaiptasia diaphana* (Ed).

All the five proteins contained the conserved S_TKc domain. Figure 2A shows the alignment of three subdomains (I, II, and VIb) of S_TKc domain for the Tssks used in the phylogenetic analysis. As seen, except for Tssk5 that has insertions in subdomain II, the other Tssk proteins are similar in length in these regions. There are two important regions in the S_TKc domain, i.e., the serine/threonine protein kinases active site and ATP-binding region. The ATP-binding region located at subdomain I and II is a glycine-rich stretch of residues, which is present in five Tssk subfamilies (Tssk1/2, Tssk3, Tssk4, Tssk6, and Tssk7). The serine/threonine protein kinases active site is 13 residues in length and exists in all six Tssk subfamilies at subdomain VIb. The indispensable lysine residue that participates in ATP binding and the conserved aspartic acid residue, which is important for the catalytic activity of the enzyme, are present in all the Tssks we investigated.

To determine which subfamily *A. irradians Tssk* genes belong to, a neighbor-joining (NJ) phylogenetic tree was constructed using the conserved S_TKc domain of Tssk proteins from animals belonging to various phyla (Figure 2B). Results showed that the five *Tssk* genes of *A. irradians* were clustered into five independent clades, of which four have been reported in vertebrates, including Tssk1/2, Tssk3, Tssk4, and Tssk5. The remaining one (Tssk7) clustered with Tssks from other mollusks and arthropods. No Tssk6 was found in the scallop or other invertebrates.

### Temporal Expression of *Argopecten irradians Tssk* Genes in Adult Gonads

**Figure 3:**
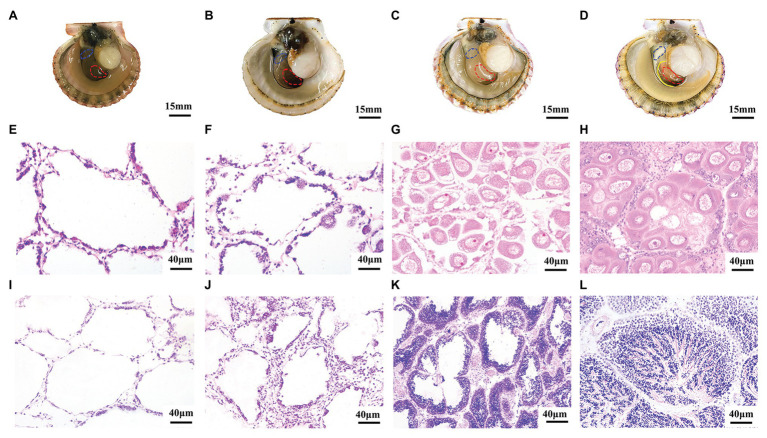
Morphological observation of four developmental stages during gametogenesis of *A. irradians*. **(A–D)** The anatomical pictures of bay scallops at resting stage, proliferative stage, growing stage, and maturation stage, respectively. The left shell valve was removed, with anterior towards left. The solid yellow line indicates the boundary of ovary and testis, and the red and blue dotted lines mark the ovary and testis we collected, respectively. **(E–H)** Represent the histological observation of ovaries at the corresponding stages. **(I–L)** Represent the histological observation of testes at the corresponding stages.

In order to obtain the expression patterns of *A. irradians Tssk* genes in the gonads, we first determined the developmental stages of testes and ovaries (Figure 3). Like other bivalves, the gonads of *A. irradians* can be classified into four stages: resting stage, proliferative stage, growing stage, and maturation stage. Although *A. irradians* is a simultaneous hermaphrodite with distinct testis and ovary, the male and female portion could only be judged visually at the maturation stage when the testis is white and the ovary is orange (Figures 3A–D). Based on histological analysis, the follicle was empty at the resting stage, containing a single layer of follicle cells and a few spermatogonia or oogonia (Figures 3E,I). When the gonad entered the proliferative stage, germ cells began to increase and spermatocytes or oocytes appeared (Figures 3F,J). At growing stage, there were multiple layers of germ cells in the follicle. Spermatids and mature oocytes showed up in the testis and ovary, respectively (Figures 3G,K). When the gonads developed to maturation stage, the follicle was filled with mature oocytes or radially arranged spermatozoa (Figures 3H,L).

**Figure 4:**
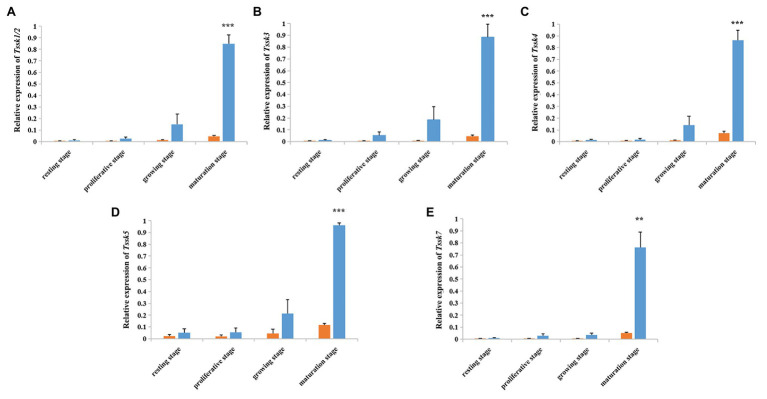
Temporal expression profile of five *A. irradians Tssk* genes in ovaries (orange) and testes (blue) during the gametogenic cycle. **(A–E)** Represent Tssk1/2, Tssk3, Tssk4, Tssk5, and Tssk7, respectively. Significant differences: ^**^p < 0.01; ^***^p < 0.001.

According to the qRT-PCR assay (Figure 4), all the five *Tssk* genes of *A. irradians* displayed a testis-predominant expression pattern. The highest expression level was found at the maturation stage, followed by the growing stage. Significant difference (*p* < 0.01) between the ovaries and testes can be observed at the maturation stage for all five *Tssk* genes. This interesting expression pattern suggests Tssk family may play an important role at the late stages of spermatogenesis in *A. irradians*.

### Localization of the *Argopecten irradians Tssk* Genes in Mature Testes

In order to confirm the location of *Tssks* in the testis, *in situ* hybridization was conducted. Here, mature testis was used for hybridization due to the following two reasons: (1) all the *Tssk* genes peaked at the maturation stage based on qRT-PCR assay and (2) the diverse types of germ cells can be found in the mature testis including spermatogonia, spermatocytes, spermatids, and spermatozoa.

**Figure 5:**
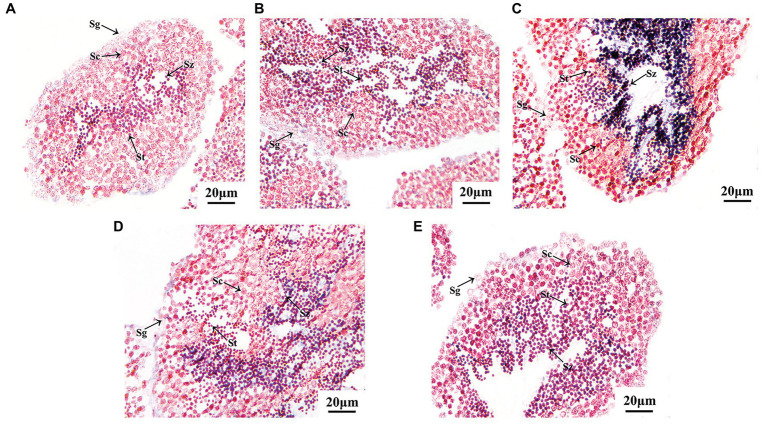
Localization of *A. irradians Tssk* genes in mature testes by *in situ* hybridization with the anti-sense probes. **(A–E)** Represent Tssk1/2, Tssk3, Tssk4, Tssk5, and Tssk7, respectively. Sg, spermatogonium; Sc, spermatocyte; St, spermatid; Sz, spermatozoon.

As shown in Figure 5, all the *Tssk* genes of *A. irradians* displayed a similar expression pattern, with strong signals in spermatids and spermatozoa, and no obvious signals in spermatogonia or spermatocytes. This result consists with the high expression of *Tssks* in mature testis as observed in qRT-PCR assay, because spermatids and spermatozoa could only be found at the late stages of spermatogenesis. No signal was detected in the ovary or testis with the sense probes (not shown).

## Discussion

In present study, five *Tssk* genes were identified in the scallop *A. irradians*, which is comparable with that of vertebrates (six in mouse and five in human and porcine; Li et al., 2011; Wang et al., 2015a). It suggests no significant duplication events occurred for Tssk family during the evolution of bilateria. Specifically, a single copy of Tssk3, Tssk4, and Tssk5 were found in vertebrates and invertebrates. But difference exists for the other subfamilies (Tssk1/2, Tssk6, and Tssk7). For Tssk1/2, a single member was found in invertebrates, in contrast to multiple members (2–3) in vertebrates, suggesting that duplication events occurred for this specific subfamily. Presence of Tssk1/2, Tssk3, Tssk4, and Tssk5 in both protostomes and deuterostomes suggests that these subfamilies may already exist in the ancestors of bilaterally symmetrical animals, rather than in the ancestor of all amniotes (birds, reptiles, and mammals) after diversification of amphibians and amniotes as previously stated (Shang et al., 2013). Tssk6 is absent in *A. irradians* as well as other invertebrates, which is expectable, because Tssk6 was considered to have a late evolutionary origin that restricts to vertebrates (Spiridonov et al., 2005). The previously unknown clade Tssk7 was present in mollusks and arthropods, but not in deuterostomes, suggesting Tssk7 is unique to protostomes and could be a new member of Tssk family.

In mollusks, previous studies on Tssk genes have focused on Tssk1/2, specifically on the gene structure and expression pattern. The four exon/three intron structure of Tssk1/2 of *A. irradians* is similar to its orthologs from Mollusca and Branchiopoda, such as *Lottia gigantea*, *M. yessoensis*, and *L. anatine* (Kim et al., 2019), suggesting that this multi-intron structure of Tssk1/2 may already be present in the lophotrochozoan ancestor. In *A. irradians*, *Tssk1/2* showed the highest expression in mature testis. Similarly, testicular levels of *Tssk1* were also affected by reproductive cycle in the pen shell and abalone, with the highest expression at the ripe/spent stage (Li et al., 2016; Kim et al., 2019). These results suggest a conserved role of Tssk1/2 in male germ cell development in mollusks. Although expression of *Tssk1/2* in the testis has been reported, its spatial localization remains unknown. According to our results, *Tssk1/2* distributes in spermatids and spermatozoa of *A. irradians*, which is in accordance with our assumption of its expression in the late-phase male germ cells based on the qRT-PCR assay. This expression pattern is also similar with that of *Tssk1* and *Tssk2* in vertebrates (Li et al., 2011; Salicioni et al., 2020), suggesting that the role of Tssk1/2 in spermatogenesis could be conserved in bilaterian animals.

Tssk5 of *A. irradians* possesses the S_TKc domain, in which a conserved serine/threonine protein kinases active site was found. However, it does not seem to have the ATP-binding region. This structure is similar to Tssk5 of other organisms. Based on previous research on Tssk5 in mammals, it could be a pseudogene in primates (Zhang et al., 2010) and might not perform as an active kinase in mouse (Li et al., 2011). But just like other Tssk members, Tssk5 have the conserved lysine residue (Figure 2) that is involved in ATP binding. Moreover, *Tssk5* has the same expression pattern in male testis as other Tssk members in the bay scallop *A. irradians* (this study), Yesso scallop *P. yessoensis*, and clam *Tridacna squamosa* (Li et al., 2020). Considering the presence of key amino acids and the similar expression pattern with other Tssk members, we assume that Tssk5 may be a functional gene in mollusks.

According to the qRT-PCR assay and *in situ* hybridization results, all the five *Tssk* transcripts displayed the same expression pattern in the scallop testis. The accumulation of Tssks in spermatids or spermatozoa is similar to vertebrates (Li et al., 2011; Salicioni et al., 2020), suggesting that the involvement of Tssks in testis development could be conserved across taxa. However, it raises questions such as why it’s necessary to have five Tssks in the scallop and what’s the functional difference between these genes. Although we cannot answer yet, studies in mammals may give us some hints. For example, Tssk1 and Tssk4 have an acrosomal and flagellar localization in mouse sperm, while Tssk2 and Tssk6 were present only in a specific sperm head compartment rich in F-actin (Li et al., 2011; Salicioni et al., 2020). Difference in subcellular localization of Tssks in mouse suggests that each Tssk member plays a distinct role in sperm function. Furthermore, the role of Tssks has been explored using genetic models in mouse. For example, the targeted deletion of both *Tssk1* and *Tssk2* genes resulted in a loss of the chromatoid body in mouse spermatids, indicating a role of Tssk1/2 in postmeiotic cytodifferentiation of spermatids (Shang et al., 2010). Therefore, subsequent immunolocalization experiments, together with an attempt of Tssk knock-out by CRISPR-Cas9 would undoubtedly assist in elucidating the specific function of each Tssk in spermiogenesis, especially the novel member Tssk7.

Although Tssks are regarded as testis-specific genes, we observed slight expression of *Tssks* in the ovaries, and the expression level also peaked at the maturation stage. Interestingly, the expression of Tssks outside the testis is not unique to the bay scallop. *Tssk1/2* was reported to be expressed in the immature gonad and ovary of a closely related hermaphroditic scallop *A. purpuratus* (Boutet et al., 2008), and low expression of Tssks in human tissues other than testis has been reported as well (Hao et al., 2004). These studies suggest a potential function of Tssks in the tissues outside of testis, which is worthy of further investigation.

## Conclusion

In this study, a total of five *Tssk* genes were identified from *A. irradians*, including a new member Tssk7 that has never been reported before. The spatiotemporal expression of Tssks indicated that all of them were almost exclusively expressed postmeiotically in the testis, suggesting that they may play pivotal roles in spermiogenesis in the scallop. To our knowledge, this study represents the first comprehensive analysis of Tssk family in mollusks. Further studies on the function of each Tssk member would assist in better understanding of sperm development in mollusks and possibly contribute to the knowledge of male sterility in some bivalves.

## Data Availability Statement

The datasets presented in this study can be found in online repositories. The names of the repository/repositories and accession number(s) can be found in the article/supplementary material.

## Author Contributions

LZ, SWa, and ZB conceived and designed the experiments. XX, HW, TL, and LL performed the experiments. QX provided the experimental scallop materials. XX, TL, and LL collected the samples. XX, YL, and SWu participated in data analysis. XX and LZ wrote the manuscript. All authors contributed to the article and approved the submitted version.

### Conflict of Interest

The authors declare that the research was conducted in the absence of any commercial or financial relationships that could be construed as a potential conflict of interest.

## Footnotes

^1^https://www.ncbi.nlm.nih.gov/

^2^https://www.uniprot.org/

^3^https://www.ncbi.nlm.nih.gov/orffinder/

^4^http://smart.embl.de/

^5^http://ibs.biocuckoo.org

^6^https://web.expasy.org/compute_pi/

^7^https://www.ncbi.nlm.nih.gov/Structure/cdd/wrpsb.cgi

^8^https://prosite.expasy.org/
